# Survival of endometrial cancer patients in Germany in the early 21st century: a period analysis by age, histology, and stage

**DOI:** 10.1186/1471-2407-12-128

**Published:** 2012-03-30

**Authors:** Tianhui Chen, Lina Jansen, Adam Gondos, Meike Ressing, Bernd Holleczek, Alexander Katalinic, Hermann Brenner

**Affiliations:** 1 Institute of Social and Family Medicine, School of Public Health, Zhejiang University, Hangzhou 310058, China; 2 Division of Clinical Epidemiology and Aging Research, German Cancer Research Center, Heidelberg, Germany; 3 Cancer Registry of Rhineland-Palatinate, Institute of Medical Biostatistics, Epidemiology and Informatics, University Medical Center, Johannes Gutenberg University Mainz, Mainz, Germany; 4 Saarland Cancer Registry, Saarbrücken, Germany; 5 Institute for Clinical Epidemiology, Cancer Registry of Schleswig-Holstein, University of Lübeck, Lübeck, Germany; 6 Institute of Social and Family Medicine, School of Medicine (Post box 25#), Zhejiang University, Yuhangtang Road 866, Hangzhou 310058, Zhejiang Province, China; 7 Members of the GEKID Cancer Survival Working Group are listed in the acknowledgements

**Keywords:** Endometrial cancer, Survival, Germany, Cancer registries, Population based, Period analysis

## Abstract

**Background:**

Population-based studies on endometrial cancer providing survival estimates by age, histology, and stage have been sparse. We aimed to derive most up-to-date and detailed survival estimates for endometrial cancer patients in Germany.

**Methods:**

We used a pooled German national dataset including data from 11 cancer registries covering a population of 33 million people. 30,906 patients diagnosed with endometrial cancer in 1997-2006 were included. Period analysis was performed to calculate 5-year relative survival (RS) in 2002-2006. Trends in survival between 2002 and 2006 were examined using model-based period analysis. Age-adjustment was performed using five age groups (15-44, 45-54, 55-64, 65-74, and 75+ years).

**Results:**

Overall, age-adjusted 5-year relative survival in 2002-2006 was 81%. A moderate age gradient was observed, with 5-year RS decreasing from 90% in the age group 15-49 years to 75% in the age group 70+ years. Furthermore prognosis varied strongly by histologic subtypes and stage, with age-adjusted 5-year RS ranging from 43% (for sarcoma) to 94% (for squamous metaplasia), and reaching 91% for localized, 51% for regional, and 20% for distant stage. Except for age group 65-74 years, no significant improvement in survival was seen during the recent 5-year period under investigation.

**Conclusion:**

In this comprehensive population-based survival analysis of patients with endometrial cancer from Germany, prognosis of endometrial cancer moderately varied by age, and strongly varied by histology and stage. While prognosis is rather good overall, further improvement in 5-year relative survival of endometrial cancer patients has been stagnating in the early 21^st ^century.

## Background

According to estimates by the International Agency for Research on Cancer (IARC) [1], cancer of the corpus uteri (commonly called endometrial cancer) ranks as the 2^nd ^most common gynecological cancer (behind cervical cancer) worldwide with 287,000 new cases diagnosed in 2008, and ranks as the 3^rd ^most common cause of gynecologic cancer death (behind cervical and ovarian cancer) worldwide with 74,000 deaths in 2008. The burden of endometrial cancer is more severe in developed countries, including Germany, where endometrial cancer ranks as the 1^st ^most common gynecological cancer with 10,776 new cases diagnosed in 2008, and ranks as the 3^rd ^most common cause of gynecologic cancer deaths (behind ovarian and cervical cancer) with 1,760 deaths in 2008 [2].

Endometrial cancer predominantly occurs in postmenopausal women [3] and is known to be related to obesity and the reproductive factors parity and age at birth [4,5]. The worldwide increase in obesity and decrease in fertility suggest that incidence of endometrial cancer will continue to rise [4], indicating that endometrial cancer will become a substantial public health problem in the future.

Endometrial cancer is generally associated with a favorable prognosis as most patients are diagnosed at early stages, most likely due to frequent postmenopausal vaginal bleeding, which enables timely diagnosis and commencement of therapy. According to estimates by the EUROCARE-4 study, age-adjusted 5-year relative survival (RS) estimates reached 76% in Europe in 1995-1999, ranging from 68% in Portugal to 84% in Sweden [6]. Nevertheless, patients diagnosed at advanced stage have poor prognosis [7-9] and survival differs substantially for histologic types [8,10]. Population-based survival data by histology have been sparse worldwide, particularly for Germany as they mostly relied on data from the Saarland Cancer Registry in the past, covering only 1.3% of the total German population [11].

In this article we provide detailed (stratified by age, histology, and stage) population-based survival estimates of endometrial cancer patients in Germany based on a pooled German national database from 11 population-based cancer registries, covering 33 million inhabitants. Furthermore, we employed standard and model-based period analysis [12-15] to provide most up-to-date estimates and trends of survival in the early 21^st ^century.

## Methods

### Database

This analysis is based on a pooled German national dataset described in detail previously [16]. Briefly, data from 11 population-based German cancer registries (covering a population of 33 million residents, i.e., 40% of the German population) with estimated completeness > 90% in the period 2004-2006 were combined. Patients aged 15 years or older and diagnosed with malignant tumors during 1997-2006 were included. Follow up with respect to vital status was performed until the end of 2006.

The current analysis focuses on patients diagnosed with endometrial cancer (ICD-10 code: C54). According to the International Classification of Diseases for Oncology (ICD-O-3) [17] and the Surveillance, Epidemiology and End Results (SEER) Survival Monograph published in 2007 [8], cancers were grouped into four major histologic groups: adenocarcinoma, carcinoma not otherwise specified (NOS), sarcoma and other specified types, and others (mixture). Adenocarcinomas were further divided into 8 subtypes (adenocarcinoma NOS, papillary, clear cell, squamous metaplasia, mucinous, adenosquamous, endometrioid, and other adenocarcinoma). For more details about histology and morphology codes and their frequencies in the analyzed dataset please refer to the Appendix.

Stage of disease at diagnosis was defined according to the recommendation of European Network of Cancer Registries (ENCR) [18], using a variable indicating grouped clinical stage with four categories, i.e., localized (tumors localized/with local spread), regional (tumors with regional spread), distant (advanced cancer), and unknown.

### Statistical analysis

Period analysis [12,13] was used to derive 5-year relative survival (RS) estimates for 2002-2006. Period analysis provides more up-to-date survival estimates than traditional cohort-based survival analysis by focusing exclusively on survival experience during the most recent time period for which data are available. This is achieved by left truncation of observations at the beginning of the period of interest in addition to right censoring of observations at its end. It has been shown by extensive empirical evaluations that period estimates of 5-year survival for a specific period closely predict 5-year survival later observed for patients diagnosed in that period [19,20]. Relative survival was calculated as the ratio of the observed survival in the group of endometrial cancer patients divided by the expected survival of a comparable group from general population [21]. Expected survival was derived from life tables for the population of Germany stratified by age, sex, calendar period and federal states, using the Ederer II method [22]. Reporting of relative survival, which has been the standard method used in population based cancer survival analyses for several decades, was preferred over reporting of a recently proposed estimate of net survival [23] for consistency and comparability with other published data. Five-year RS was calculated by histologic subtypes for 3 major age groups (15-49, 50-69, and 70+) in analogy with the SEER Survival Monograph published in 2007 [8]. RS estimates were not reported if the standard errors exceeded 5 percent units. In addition, age-adjusted 5-year RS was calculated for patient subgroups defined by histology and stage.

In addition to "standard" period analysis [13], model-based period analysis [14,15] was employed to assess recent trends within the 2002-2006 period. Model-based period analysis can provide survival estimates that are as up-to date as those from standard period analysis and at the same time much more precise than the latter. In addition, this approach allows testing for trends over time. The method has been described in detail elsewhere [14]. Briefly, age group-specific numbers of patients at risk and of deaths by year of follow-up for each single calendar year between 2002 and 2006 were computed. The numbers of deaths were then modeled as a function of age group at diagnosis (entered as categorical variable), the year of follow-up (categorical variable) and the calendar year (continuous variable) by Poisson regression with the logarithm of the person-years at risk as offset [14]. Model-based estimates of 5-year RS for the first (2002) and last year (2006) of the period and a p-value for the trend in RS between 2002 and 2006 were derived. A p value of 0.05 was used as the level of significance for trend tests. Standard errors of the model-based 5-year RS estimates were calculated using the delta method as previously described [14].

Age-adjustment for standard period analysis was done by deriving weighted averages of age-specific 5-year RS estimates, using weights of five age groups (15-44, 45-54, 55-64, 65-74, and 75+ years) according to the International Cancer Survival Standards [24].

All calculations were performed with the SAS statistical software package (version 9.2, SAS Institute Inc., Cary, North Carolina), using special macros for the period analysis as described in detail elsewhere [14,25].

## Results

The basic characteristics of the dataset used in the current period analysis for endometrial cancer patients diagnosed in Germany from 1997 to 2006 are presented in Table 1. After exclusion of 1,355 patients (4.2%) notified by death certificate only (DCO) or by autopsy only, and 2 patients without dates of diagnosis or death, 30,906 cases with a median age at diagnosis of 67 years were included in the analysis. 99% of cases were microscopically confirmed. Although all registries provided staging information, there were 13,456 cases (44% of total) with complete stage information.

**Table 1:** Description of the dataset used in period survival analysis for endometrial cancer patients diagnosed in Germany, 1997-2006

Registry	Population covered (Million)	Count (%)	Stage information available	Median age	Microscopically Confirmed (%)
Bavaria	8.13	4,893 (15.8)	Yes	67	99.9
Brandenburg	2.55	3,223 (10.4)	Yes	66	99.7
Bremen	0.66	882 (2.9)	Yes	67	99.6
Hamburg	1.75	1,359 (4.4)	Yes	67	98.0
Mecklenburg-Vorpommern	1.69	2,204 (7.1)	Yes	66	99.7
Lower Saxony	7.98	4,942 (16.0)	Yes	67	97.9
North Rhine-Westphalia	2.62	2,190 (7.1)	Yes	67	98.6
Rhineland-Palatinate	0.52	560 (1.8)	Yes	68	99.1
Saarland	1.04	1,577 (5.1)	Yes	68	99.9
Saxony	4.25	7,096 (23.0)	Yes	68	99.6
Schleswig-Holstein	1.85	1,980 (6.4)	Yes	68	99.8
Total	33.04	30,906 (100)	13,456 (44%)	67	99.3

Age-adjusted and age group-specific 5-year RS estimates for the time period 2002-2006 by histology are presented in Table 2. Overall, age-adjusted 5-year RS in 2002-2006 was 81.0%. Prognosis strongly varied by histology, with age-adjusted 5-year RS ranging from 43.1% (sarcoma) to 94.3% (squamous metaplasia). Age-adjusted 5-year RS estimates > 80% were observed for endometrioid adenocarcinomas (the largest group, accounting for 44.2% of all cases), adenocarcinoma NOS, squamous metaplasia, and mucinous cancers. Overall, a moderate age gradient was observed, with 5-year RS decreasing from 90.0% in age group 15-49 years to 74.8% in age group 70+ years. Patients with uterine sarcoma had a much stronger age gradient, with 5-year RS ranging from 75.7% in age group 15-49 years to 31.7% in age group 70+ years. Furthermore, patients with uterine sarcoma had the worst prognosis in all age groups. By contrast, patients with squamous metaplasia had excellent prognosis, with 5-year RS estimates > 90%, in all age groups.

**Table 2:** Age-adjusted and age group-specific 5-year relative survival (RS) for the period 2002-2006 by histologic subtypes of endometrial cancer patients diagnosed in Germany, 1997-2006^a^

Histologic subtypes	Overall^b^		Age groups					
			
			**15**-**49 years**		**50**-**69 years**		70+ years	
	
	Count (%^c^)	RS (SE)	Count (%)	RS (SE)	Count (%)	RS (SE)	Count (%)	RS (SE)
**Overall**	30,906 (100)	81.0 (0.4)	1,949 (6)	90.0 (1.0)	15,995 (52)	84.8 (0.4)	12,962 (42)	74.8 (0.8)
**Adenocarcinoma**	28,273 (91.5)	83.9 (0.4)	1,578 (6)	92.6 (1.0)	14,748 (52)	87.6 (0.4)	11,947 (42)	77.8 (0.8)
Adenocarcinoma, NOS	10,427 (33.7)	84.7 (0.7)	606 (6)	93.5 (1.5)	5,353 (51)	88.7 (0.7)	4,468 (43)	78.1 (1.3)
Papillary	1,141 (3.7)	68.4 (2.4)	50 (4)		547 (48)	77.2 (2.7)	544 (48)	57.3 (3.8)
Clear cell	330 (1.1)	62.0 (4.3)	8 (2)		140 (43)		182 (55)	
Squamous metaplasia	686 (2.2)	94.3 (2.5)	65 (9)	95.2 (3.9)	375 (55)	92.3 (2.4)	246 (36)	94.1 (4.9)
Mucinous	268 (0.9)	85.2 (4.1)	12 (5)		130 (48)	93.4 (3.9)	126 (47)	
Adenosquamous	857 (2.8)	75.0 (2.5)	57 (7)		453 (53)	80.1 (2.9)	347 (40)	64.3 (4.8)
Endometrioid	13,650 (44.2)	86.0 (0.7)	738 (5)	93.7 (1.4)	7,323 (54)	88.8 (0.6)	5,589 (41)	81.3 (1.3)
Other adenocarcinoma^d^	914 (2.9)	68.3 (2.5)	42 (4)		427 (47)	70.5 (3.3)	445 (49)	57.2 (4.4)
**Carcinoma NOS**	619 (2.0)	60.4 (3.3)	42 (7)		272 (44)	69.1 (4.4)	305 (49)	
**Sarcoma**	1,682 (5.4)	43.1 (1.8)	287 (17)	75.7 (3.8)	834 (50)	47.6 (2.4)	561 (33)	31.7 (3.3)
**Others (mixture)^e^**	332 (1.1)	47.3 (4.2)	42 (13)		141 (42)		149 (45)	

Abbreviations: NOS = not otherwise specified; RS = point estimates of relative survival (%); SE = standard error (percent units);

^a ^unstable point estimates of RS (SE > 5 percent units) were not reported;

^b ^age-adjusted 5-year relative survival;

^c ^% of histologic subtypes;

^d ^including papillary serous adenocarcinomas (70 cases);

^e ^including other specified carcinomas (42 cases).

Table 3 shows age-adjusted 5-year RS by histology and stage. Among the restricted dataset with complete information on stage 13,456 cases (44% of total), overall 86% cases were diagnosed at localized stage and only 7% were diagnosed at distant stage. Prognosis strongly varied by stage, with age-adjusted 5-year RS reaching 91.2% for localized stage, 50.5% for regional stage, and 19.8% for distant stage. The strong gradient in prognosis by tumor stage was consistently seen within major histologic subtypes. A less favorable prognosis and lower proportion in localized stage were noted for sarcoma.

**Table 3:** Age-adjusted 5-year relative survival (RS) for the period 2002-2006 by histologic subtypes and stage for endometrial cancer patients diagnosed in Germany, 1997-2006

Histologic subtypes	Stage									
	
	With complete stage information								No stage information	
			
	Overall		Localized		Regional		Distant			
	
	N (%^b^)	RS (SE)	N (%^c^)	RS (SE)	N (%^c^)	RS (SE)	N (%^c^)	RS (SE)	N (%^d^)	RS (SE)
**Overall**	13,456 (100)	81.0 (0.4)	11,634 (86)	91.2 (0.7)	814 (6)	50.5 (3.1)	1,008 (7)	19.8 (2.0)	17,450 (56)	79.5 (0.6)
**Adenocarcinoma**	12,630 (94)	83.9 (0.4)	11,097 (88)	92.2 (0.7)	736 (6)	50.7 (3.3)	797 (6)	20.0 (2.1)	15,643 (55)	83.3 (0.6)
Adenocarcinoma NOS	4,161 (31)	84.7 (0.7)	3,652 (88)	93.7 (1.1)	221 (5)		288 (7)	18.4 (3.3)	6,266 (60)	83.9 (0.9)
Papillary/clear cell	664 (5)	68.0 (2.2)	523 (79)	83.0 (3.0)	61 (9)	14.0 (4.9)	80 (12)	10.4 (5.0)	807 (55)	70.1 (2.7)
Mucinous/Squamous metaplasia	440 (3)	91.1 (2.2)	388 (88)	96.9 (3.2)	28 (6)		24 (5)		514 (54)	90.8 (2.9)
Adenosquamous	443 (3)	75.0 (2.5)	367 (83)	86.1 (3.7)	35 (8)		41 (9)		414 (54)	72.9 (3.7)
Endometrioid	6,499 (48)	86.0 (0.7)	5,841 (90)	92.9 (1.0)	344 (5)	57.2 (4.9)	314 (5)	22.3 (3.5)	7,151 (52)	84.9 (0.9)
Other adenocarcinoma	423 (3)	68.3 (2.5)	326 (77)	76.8 (4.4)	47 (11)		50 (11)	5.6 (4.5)	491 (54)	71.6 (3.4)
**Sarcoma**	521 (4)	43.1 (1.8)	350 (67)	61.8 (4.4)	48 (9)		123 (24)	13.7 (4.0)	1,161 (69)	40.1 (2.1)

Abbreviations: NOS = not otherwise specified; RS = point estimates of relative survival (%); SE = standard error (percent units);

^a ^unstable point estimates of RS (SE > 5 percent units) were not reported;

^b ^% of histology;

^c ^% of those with complete stage information;

^d ^% of cases without stage information.

Model-based age-specific 5-year RS estimates for 2002 and 2006 are shown in Table 4. Overall, there was little change in prognosis between 2002 and 2006, with age-adjusted 5-year RS reaching 80.1% in 2002 and 81.3% in 2006. Likewise, similar 5-year RS estimates were observed for most age subgroups. Only for age group 65-74 years a significant improvement in prognosis was seen, with 5-year RS increasing from 78.6% in 2002 to 82.4% in 2006 (change of 3.8 percent units, p = 0.034). In addition, for both calendar years, a similarly moderate age gradient in 5-year RS was observed.

**Table 4:** Model-based age group-specific 5-year relative survival (RS) in 2002 and 2006 for endometrial cancer patients diagnosed in Germany,1997-2006

Age	2002 (1)		2006 (2)		Change of RS (2)-(1)^a^	P-value^b^
			
	Count (%)	RS (SE)	Count (%)	RS (SE)		
**Age groups**						
15-44 years	94 (2)	92.8 (2.0)	114 (3)	91.3 (2.4)	-1.5	0.691
45-54 years	398 (10)	87.2 (1.5)	412 (11)	88.6 (1.3)	1.4	0.551
55-64 years	1,028 (27)	86.1 (0.9)	887 (23)	85.4 (1.0)	-0.7	0.670
65-74 years	1,236 (33)	78.6 (1.2)	1,368 (36)	82.4 (1.0)	3.8	0.034
75+ years	1,047 (28)	72.0 (1.6)	1,028 (27)	71.8 (1.6)	-0.2	0.952
**Overall **^c^	3,803 (100)	80.1 (0.7)	3,809 (100)	81.3 (0.6)	1.2	0.256

Abbreviations: RS = point estimates of relative survival (%); SE = standard error (percent units);

^a ^difference in percent units;

^b ^the differences between RS estimates were tested using model-based period analysis;

^c ^age-adjusted.

Table 5 shows model-based age-adjusted 5-year RS estimates in 2002 and 2006 by histology and stage. For the 5-year period between 2002 and 2006, a trend towards improvement in survival was seen for most subgroups, but none of these changes was statistically significant. A reverse trend was even observed for uterine sarcomas.

**Table 5:** Model-based age-adjusted 5-year relative survival in 2002 and 2006 by histology and stage for endometrial cancer patients diagnosed in Germany, 1997-2006

Histology	Stage^a^	2002 (1)		2006 (2)		Change of RS (2)-(1)	P-value^b^
				
		Count	RS (SE)	Count	RS (SE)		
**Adenocarcinoma**		3,511	82.6 (0.7)	3,495	84.5 (0.6)	1.9	0.066
Adenocarcinoma, NOS		1,526	84.7 (1.0)	700	84.3 (1.1)	-0.3	0.848
Papillary/clear cell^c^		322	67.0 (2.7)	186	68.9 (2.6)	1.9	0.643
Mucinous/squamous/ adenosquamous		228	80.5 (3.2)	157	85.2 (2.5)	4.7	0.265
Endometrioid		1,435	85.7 (0.9)	2,452	85.8 (0.9)	0.1	0.961
**Sarcoma**		199	48.6 (2.6)	223	39.7 (2.6)	-8.9	0.048
**Others (mixture) **^d^		93	55.8 (4.1)	91	54.1 (4.1)	-1.7	0.789
**All**	Localized	1,409	90.5 (1.0)	1,497	91.8 (0.9)	1.3	0.362
	Regional	91	47.0 (4.2)	137	53.3 (4.0)	6.3	0.368
	Distant	116	19.4 (2.8)	146	21.4 (2.9)	2.0	0.641
	**Overall**	1,616	82.8 (0.9)	1,780	83.4 (0.9)	0.5	0.721

Abbreviations: NOS = not otherwise specified; RS = point estimate of relative survival (%); SE = standard error (percent units);

^a ^restricted to cases with complete stage information;

^b ^the differences between RS (percent units) were tested using model-based period analysis;

^c ^including other adenocarcinoma;

^d ^including carcinoma NOS.

## Discussion

In this manuscript we provide the first comprehensive population-based analysis of survival of patients with endometrial cancer from Germany available to date. Overall, age-adjusted 5-year relative survival in 2002-2006 was 81.0%. A moderate age gradient was observed, with 5-year RS decreasing from 90.0% in age group 15-49 years to 74.8% in age group 70+ years. Prognosis furthermore strongly varied by histologic subtypes and stage, with age-adjusted 5-year RS ranging from 43.1% (for sarcoma) to 94.3% (for squamous metaplasia), and reaching 91.2% for localized, 50.5% for regional, and 19.8% for distant stage. For the recent 5-year period under investigation, a trend towards improvement in survival was seen for most subgroups assessed, which was though rather modest and, except for age group 65-74 years, not statistically significant.

We found similar results when data were restricted to Saarland only. Therefore, the previous estimates of cancer survival from Germany, which were often based on Saarland alone, most likely had been representative. However, the extended database allowed much more detailed and precise estimates: For example, due to inclusion of 30,906 cases from multiple registries rather than 1,577 cases from Saarland alone, the standard error for 5-year relative survival of endometrial cancer overall decreased from 1.9% to 0.4%. Furthermore, our estimates of overall 5-year RS of 81.0% for Germany in 2002-2006 are very close to the corresponding estimate of 80.4% for the USA in 2002-2006 [16], and only slightly higher than the estimated EUROCARE-4 mean of 78.0% in 2000-2002 [26].

Our finding of no major improvement in survival for most subgroups within the 5-year period is consistent with three large population-based studies [7,15,26] which likewise showed that survival of endometrial cancer has been stagnating. For the calendar period 2000-2004, a trend towards improvement in survival was seen in 12 European countries, but reached statistical significance only for Estonia [15]. Furthermore, the EUROCARE-4 study using data from 47 European cancer registries [26] showed that survival figures remained rather stable over the calendar period 1997-2002, though improvements in survival were seen over the period 1991-1996. A study based on the SEER data from the USA also found no improvement in 5-year relative survival for the period 1998-2003 [7]. These trends are in contrast to observations for earlier periods. A steady increase in 5-year RS estimates for earlier periods had been reported, for example, for the Nordic [27] and other European countries [28-30], which could be mainly attributed to early diagnosis due to introductions of ultrasonography in the 1980s [31] and to advances in techniques of endometrial biopsy [3].

Given that survival of endometrial cancer is already rather good overall, with 5-year RS estimates exceeding 80% in 2002-2006 for Germany and the USA [16], and approaching 80% in 2000-2002 for most European countries [26], further improvement may be difficult to achieve and stagnation of survival rates may not be too surprising. So far, screening (e.g., by transvaginal ultrasound) for endometrial cancer in asymptomatic women at average risk is not recommended due to lack of sufficient evidence [32-35]. Increased rates in high-risk histologic subtypes with poor survival such as uterine sarcomas [36-38], as suggested by other studies [39], also contribute to stagnation of further improvement in overall survival rates. Poor survival of uterine sarcomas is likely due to lack of reliable diagnostic test [36,37], and to limited beneficial effect of adjuvant therapies [36].

Our finding of a statistically significant improvement in survival only for age group 65-74 years, but not for other age groups, may reflect enhanced dissemination of effective therapy to older age groups, but not to the "oldest old". A previous analysis of EUROCARE data [40] also reported that survival in age group 55-69 years improved more than that in age group 70-84 years. Our observations of a less favorable prognosis in elderly women and of a moderate age gradient are consistent with other studies [8,41,42]. The less favorable prognosis of endometrial cancer in the oldest age group might be in part attributable to comorbidities and less access to therapeutic innovations [6,43], as old patients are generally under-represented in cancer clinical trials [43]. Nevertheless, the age gradient in relative survival for patients with endometrial cancer is less pronounced than the age gradient observed for other types of cancer, and 5-year relative survival exceeded 70% even in the oldest age group.

We observed a strong variation in survival by histologic subtypes, consistent with other studies [8,42,44]. The predominant theory for the etiology of endometrial cancer is that development and progression of this cancer is strongly related to high bioavailable estrogens and/or low progesterone (unopposed estrogen hypothesis) [5,45,46]. The physiological effects of these hormones on the endometrium are transmitted by estrogen receptors (ER) and progesterone receptors (PR) [47,48]. Given that the expressions and distributions of ER (ER-a and ER-b) and PR (PR-A and PR-B) have been associated with different survival of endometrial cancer in several studies [49,52], it is plausible to assume that different profiles of ER/PR expressions and distributions in histologic subtypes of endometrial cancer may contribute to the strong variation in survival by histologic subtypes.

In agreement with previous studies (7-9, 42], we found a strong gradient in survival by stage. Early detection is the key for overall good prognosis of endometrial cancer as most women diagnosed at localized stage can be cured by surgery alone [53]. Endometrial cancer is usually diagnosed at an early stage (86% in our data) due to abnormal vaginal bleeding. Women (especially after menopause) experiencing abnormal vaginal bleeding should undergo diagnostic tests, e.g., endometrial sampling with cytological examination and measuring endometrial thickness with transvaginal ultrasound (TVU) [34,54]. Although there is insufficient evidence to recommend screening for endometrial cancer in asymptomatic women at average risk, women at very high risk of endometrial cancer should consider beginning annual testing for early detection at age 35 years [32,33].

Although information on treatment was too incomplete to be used in our data, surgery is the cornerstone of therapy for endometrial cancer regardless of stages of the disease [55]. For patients with advanced stage or with aggressive histologic subtypes, adjuvant therapy is desirable. In addition, centralized care provided by gynecologic oncologists is also an important prognostic factor for endometrial cancer, particularly for patients diagnosed at distant stage as they are more likely to undergo staging surgery and to receive adjuvant chemotherapy [56,57].

Our study has several strengths and limitations. This is the first population-based study from Germany providing survival estimates of endometrial cancer, using a pooled German national dataset with a large sample size (30,906 cases) and covering a population of 33 million people (40% of the German population). Furthermore, this study provided most up-to-date and comprehensive survival estimates of endometrial cancer in the early 21^st ^century, using the techniques of standard and model-based period analysis. Limitations are mainly related to limited staging information. Stage information was available for 44% of cases only, precluding joint stratification by stage and histology in time trend analyses. Patients with stage information were on average 2.6 years younger and had more often adenocarcinoma than carcinoma NOS, sarcoma or other (mixture) histologies, and had a slightly higher age-adjusted survival than those without stage information (81.1 (0.4)) versus 79.5 (0.6). Another limitation concerns lack of treatment information.

## Conclusion

In summary, in this first comprehensive population-based study from Germany we demonstrate that prognosis of endometrial cancer moderately varied by age, and strongly varied by histology and stage. In addition, while prognosis was found to be rather good overall, further improvement in 5-year relative survival of endometrial cancer patients has been stagnating in the early 21^st ^century except for age group 65-74 years. Progress in screening methods for early detection in asymptomatic women at high risk and dissemination of advances in therapeutic oncology to the population level, in particular for patients with biologically aggressive histologic subtypes (e.g., uterine sarcomas) and older patients, might be most important for further improvement in survival of endometrial cancer patients in the 21^st ^century.

## Competing interests

The authors declare that they have no competing interests.

## Authors' contributions

TC performed the data analysis and interpretation and wrote the manuscript. LJ contributed in data acquisition, quality control of the data and algorithms, data analysis and interpretation. AG contributed in quality control of the data and algorithms, and data interpretation. MR, BH and AK contributed in data acquisition, reviewed and edited registry abstracts. HB conceived of the study, supervised data analysis and writing of the manuscript. All authors read and approved the final manuscript.

## Grant support

This study was funded by German Cancer Aid (Deutsche Krebshilfe), grant no. 108257. Tianhui Chen's work was supported in part by the Fundamental Research Funds for the Central Universities, China. The sponsor had no role in the study design, collection, analysis, or interpretation of data.

## Appendix

Appendix I. Table 6 Description of histologic subtypes of endometrial cancer patients diagnosed in Germany, 1997-2006

**Table 6:** Description of histologic subtypes of endometrial cancer patients diagnosed in Germany, 1997-2006

Classification of histological subtypes	ICD-O-3 codes	Frequency (N)	Percent (%)
**a. Adenocarcinoma**	8050,8140-8147,8160-8162,8180-8221,8250-8506,8520-8550,8560,8570-8573,8940-8941	28,273	**91.5**

Adenocarcinoma, NOS	8140	10,427	33.7
Papillary	8050,8260	1,141	3.7
Clear cell	8310	330	1.1
With squamous metaplasia	8570	686	2.2
Mucinous	8480-8481	268	0.9
Adenosquamous	8560	857	2.8
Endometrioid	8380	13,650	44.2
Other adenocarcinoma^a^	8141-8147,8160-8162,8180-8221,8250-8259,8261-8309,8311-8379,8381-8459,8461-8479,8482-8506,8520-8550,8571-8573,8940-8941,8460	914	2.9

**b. Carcinoma NOS**	8010-8022	619	**2.0**

**c. Sarcoma and other specified types**	8680-8713,8720-8790,8800-8920,8930-8933,8950-8982,8990-8991,9000-9030,9040-9055,9060-9110,9120-9134,9141-9340,9350-9364,9380-9512,9530-9581	1,682	**5.4**

**d. Others (mixture)^b, c^**	8000-8004,8051-8130,8921,8935, 8030-8045,8150-8155,8170-8171,8230-8248,8510-8512,8561-8562,8580-8671	322	**1.1**

**In total**	8000-9581	30,906	**100**

Abbreviation: NOS = not otherwise specified;

^a ^including papillary serous adenocarcinoma (70 cases);

^b ^including other specified carcinomas (42 cases);

^c ^Missing histologies coded as 8000 were included in this subgroup.

## Pre-publication history

The pre-publication history for this paper can be accessed here:

http://www.biomedcentral.com/1471-2407/12/128/prepub
